# Primary Breast Sarcoma: A Case Report at Kuwait Cancer Center and Review of the Literature

**DOI:** 10.7759/cureus.114950

**Published:** 2026-08-21

**Authors:** Mohamed Moneer A Alsayed, Amal Yousif S Abdullah, Mohamed Mahmoud A Abokhtita, Sathyanarayanan Rajaganesan, Mitra H Behbehani Pour

**Affiliations:** 1 Surgical Oncology, Kuwait Cancer Control Center, Kuwait, KWT; 2 Clinical Pathology, Kuwait Cancer Control Center, Kuwait, KWT

**Keywords:** breast, case report, kuwait, kuwait cancer center, primary, review of literature, sarcoma

## Abstract

Breast sarcomas can be classified as primary (de novo) or, more commonly, secondary to previous radiation therapy for breast cancer or lymphoma. Primary breast sarcoma (PBS) is particularly rare (around 0.1% of all primary breast cancers and <5% of all soft tissue sarcomas) and differs from malignant phyllodes tumors with a worse prognosis. Diagnosis is by triple assessment and ruling out all the differentials (most commonly malignant phyllodes and metaplastic carcinomas) using core biopsy, but sometimes complete surgical excision is needed to confirm the tissue diagnosis. Staging and management of PBS are different from that for breast carcinoma, and they follow the guidelines of soft tissue sarcoma of the trunk and extremities. The standard of care is surgical resection with adequate negative margins (>1 cm for all sarcomas and >3 cm in angiosarcoma and leiomyosarcoma). Axillary staging can be considered only if the core biopsy diagnosis is uncertain (complete excision advised by the pathologist) or in the presence of discordance between clinical/radiological findings and fine-needle cytology of axillary lymph nodes. The role of adjuvant therapy, either radiation or systemic chemotherapy, is highly controversial, but the National Comprehensive Cancer Network (NCCN) guidelines recommend considering adjuvant radiotherapy for cases with positive margins or higher stages. In addition, there is no solid consensus about the benefit of chemotherapy, but some articles reported using it in both adjuvant and neoadjuvant settings. In conclusion, the management should be on a case-by-case basis supported by multidisciplinary team (MDT) approach. The aim of this article is to present a case of PBS with a clinical presentation of rapidly growing locally advanced breast cancer in a 32-year-old woman in the State of Kuwait. Moreover, the authors are presenting an up-to-date review of the limited literature on PBS.

## Introduction

The most common type of breast sarcoma is secondary angiosarcoma to previous radiation therapy [1]. Primary breast sarcoma (PBS) is extremely rare; the spectrum includes subtypes based on the prominent histological components, e.g., angiosarcoma, liposarcoma, leiomyosarcoma, and so forth. It is well known that the cornerstone of treatment is complete surgical resection with negative margins [1]. Management of breast sarcoma is usually based on the guidelines for soft tissue sarcomas, and it is worth mentioning that there are no specific guidelines designed for breast sarcomas as a separate entity [2]. Due to the lack of prospective studies, there is no clear indication for adjuvant therapy. According to the National Comprehensive Cancer Network (NCCN), both adjuvant radiotherapy and systemic therapy should be considered on a case-by-case approach, depending on pathological staging and the safety of surgical margins. In addition, neoadjuvant therapy may be considered for higher stages.

## Case presentation

The case in this study is a 32-year-old woman, who was BRCA mutation negative, married with no children, and has no family history of malignant diseases. She presented with a clinical picture of locally advanced, right breast cancer (simulating cT4b N1 M0 breast carcinoma). On clinical examination, there was a large palpable mass, measuring 11 x 11 cm, with a history of rapid increase in size over a short period of time (three months). The mass occupied almost the outer half of the breast and was attached to the overlying skin at multiple points, with skin redness, discoloration, and edema. In addition, right nipple retraction and multiple palpable right axillary lymph nodes were also noted.

**Figure 1:**
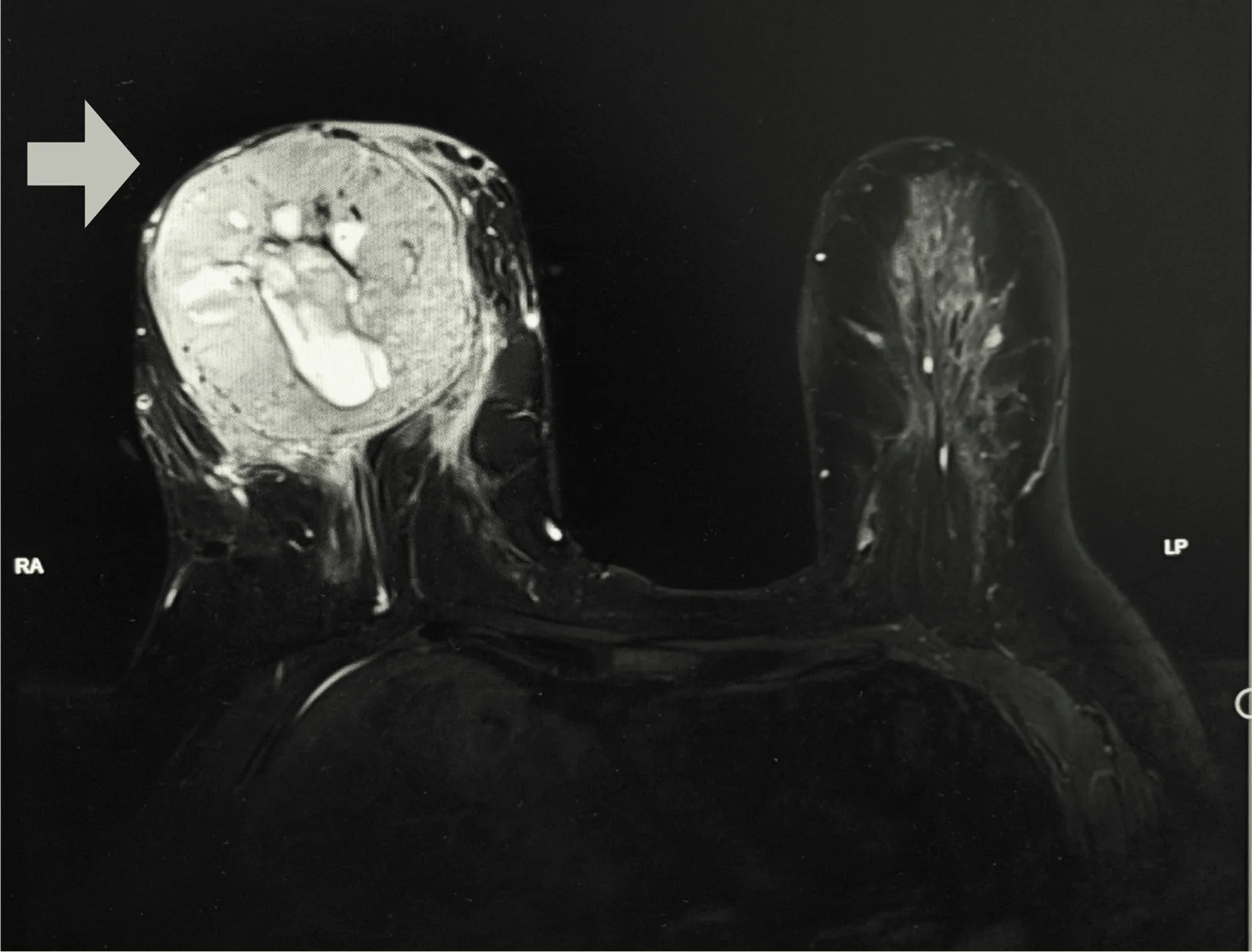
MRI: Index mass measuring 8.2 x 8 x 7.6 cm in the right breast (arrow). The mass was seen extending to all breast quadrants, inseparable from overlying skin at some points and in close proximity to the nipple (with retraction). MRI: magnetic resonance imaging.

**Figure 2:**
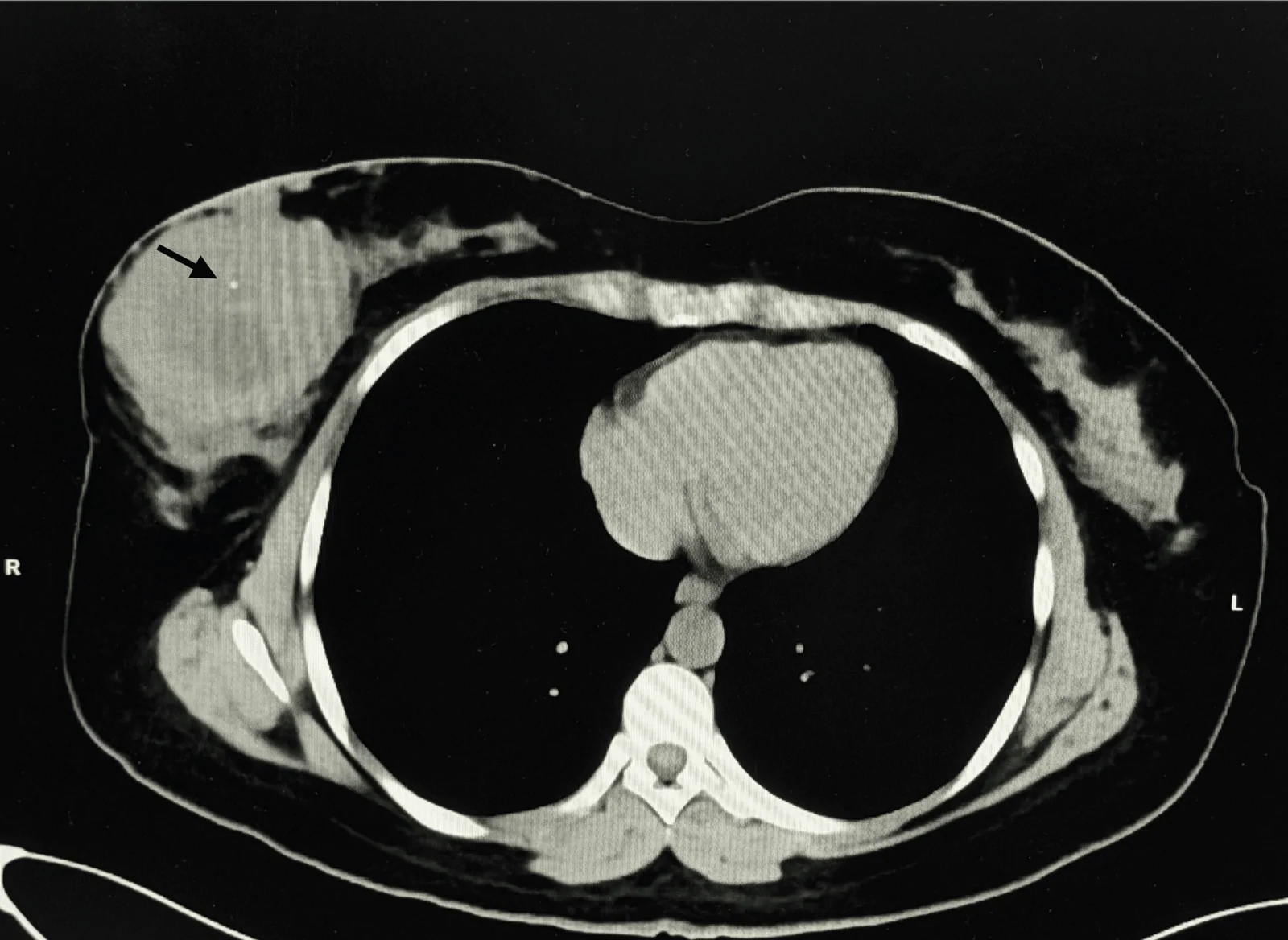
Staging CT (chest cut): Index mass in the right breast, with no evidence of lung metastasis. A core biopsy marker clip seen in the right breast (black arrow).

Dedicated breast imaging (sonomammography and breast MRI), together with staging CT (Figures 1, 2), revealed a large round-shaped mass, with irregular margins and focal areas of necrotic and hemorrhagic changes, occupying lower outer quadrant (LOQ), upper outer quadrant (UOQ), central quadrant, and some of upper inner quadrant (UIQ). The mass was located 8 mm from the nipple, with nipple retraction and multiple areas of skin involvement, in addition to multiple suspicious small masses and multiple prominent suspicious right axillary lymph nodes. 

Ultrasound-guided core biopsy revealed a "malignant spindle cell tumor with a differential diagnosis of metaplastic sarcomatoid carcinoma versus malignant phyllodes tumor." Fine-needle aspiration from right axillary lymph nodes came negative for metastasis (reactive) and a repeat came negative again. Metastatic work-up was free (M0). The case was discussed in the Multidisciplinary Team (MDT) meeting multiple times; the first-time consensus was to repeat the core biopsy, which came again with the same indefinite diagnosis, and the pathologist recommended to do a complete resection. 

After obtaining MDT consensus, the patient underwent upfront surgery in the form of a right mastectomy with sentinel lymph node biopsy due to the presence of clinicoradiological suspicious axillary lymph nodes with negative needle aspiration cytology, which came negative by frozen section. The postoperative recovery period went uneventful without complications. The final post-resection histopathological analysis revealed a "malignant undifferentiated tumor, consistent with an undifferentiated high-grade primary breast sarcoma." Margins and lymph nodes (0/3) were free. The tumor size was 8.4 x 8 x 7.5 cm. Marked stromal cellularity, atypia, and overgrowth were found together with high mitotic rate (12 mitoses per 10 high power fields). On immunohistochemistry (IHC), the tumor cells were positive for Vimentin and Bcl2, while negative for all the following: ER, PR, Her-2; pCK, CK 14, CK 8/18, P63, desmin, SMA, CD34, CD 117, and β-hCG. The Ki-67 proliferation index was 60-70%. Pathological staging was pT2 N0, consistent with the Sarcoma Staging System.

**Figure 3:**
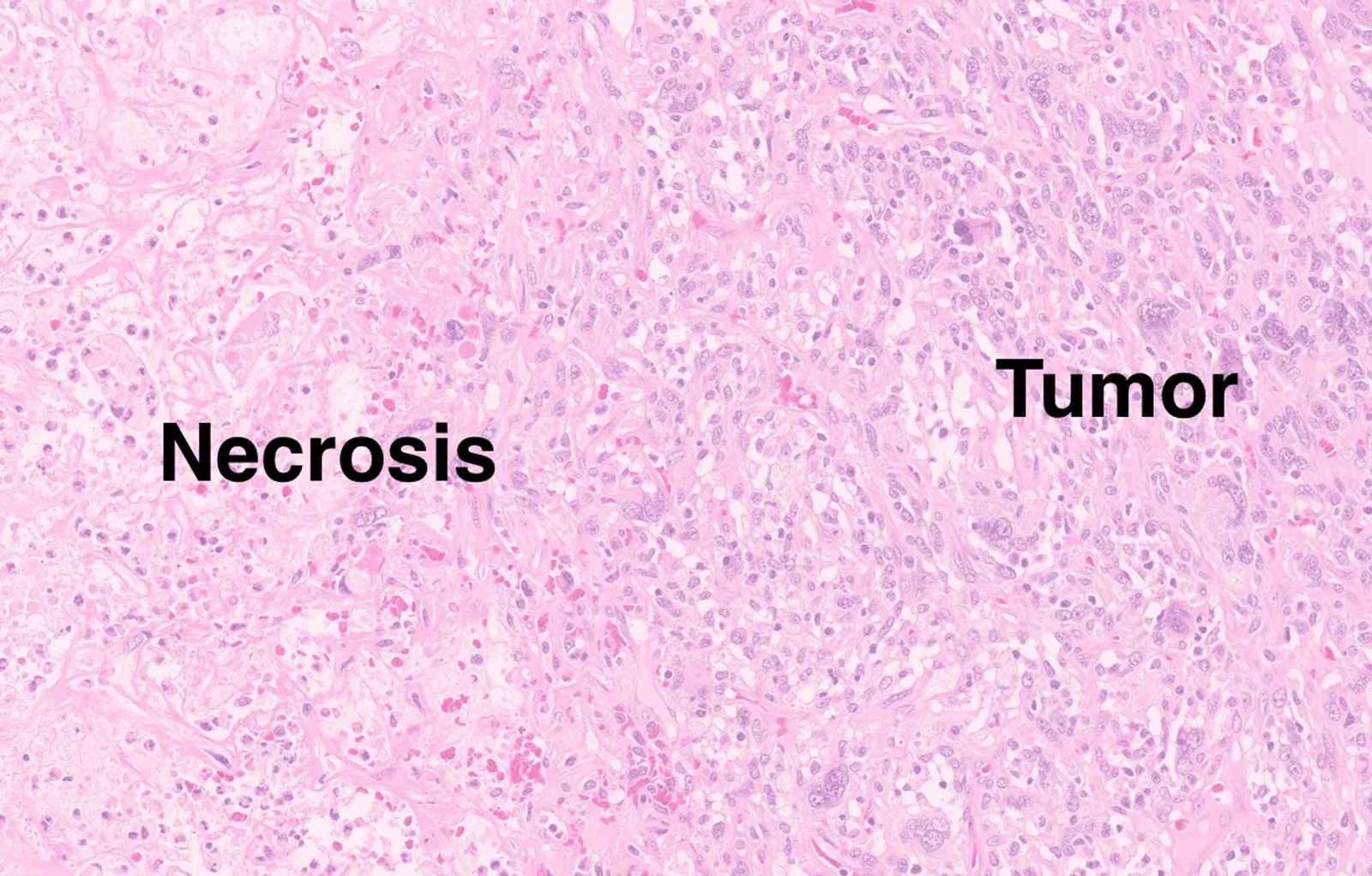
Photomicrograph of the tumor showing areas of hyperchromatic nuclei with adjacent white-colored areas of necrosis (H&E; 10×). Tumor: Hyperchromatosis, with dense staining of prominent nuclei on the right side of the image. Necrosis: White areas on the other side of the image. H&E: hematoxylin and eosin.

**Figure 4:**
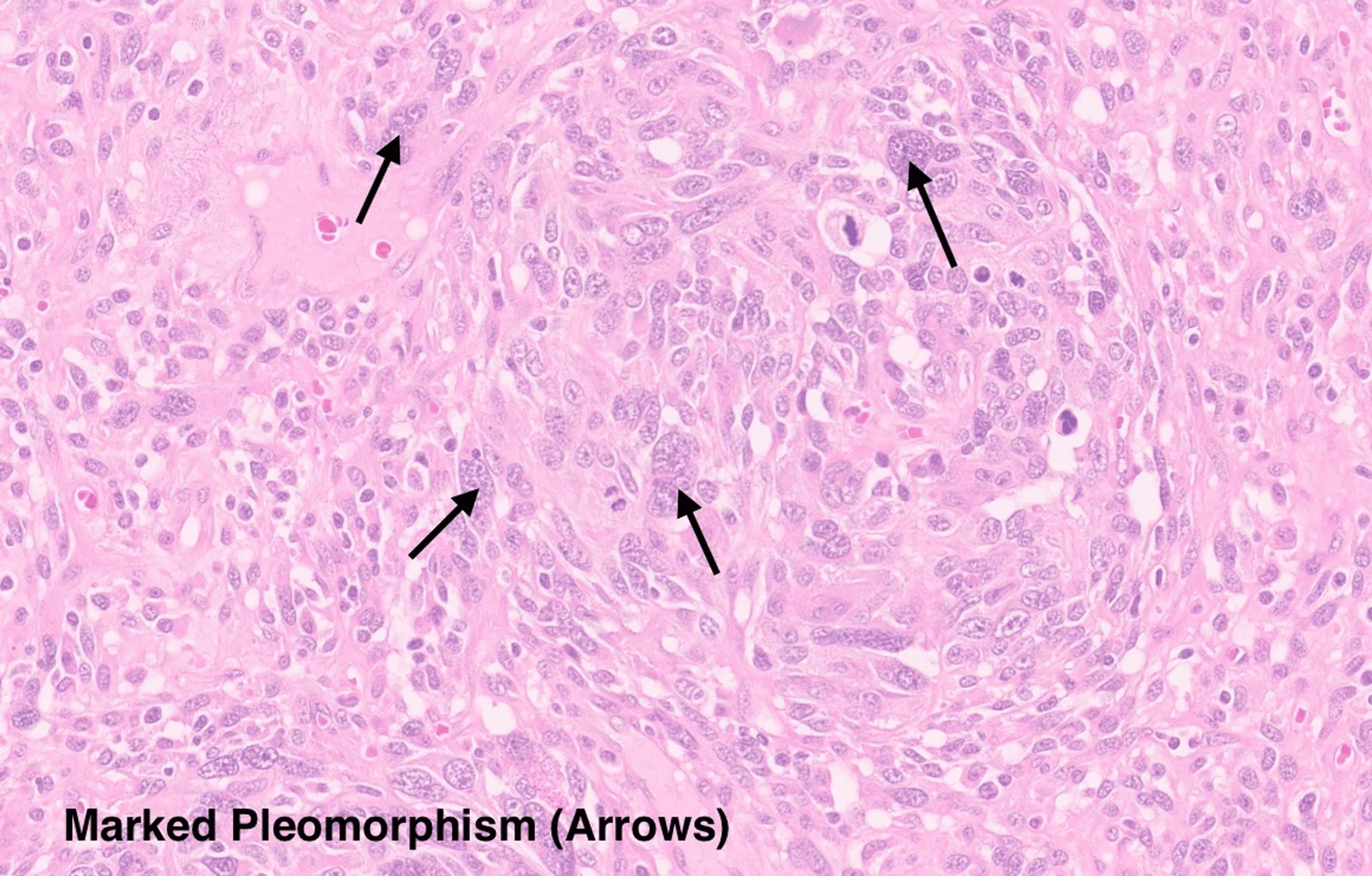
Photomicrograph of tumor giant cells with marked nuclear pleomorphism (arrows) (H&E; 20×). H&E: hematoxylin and eosin.

**Figure 5:**
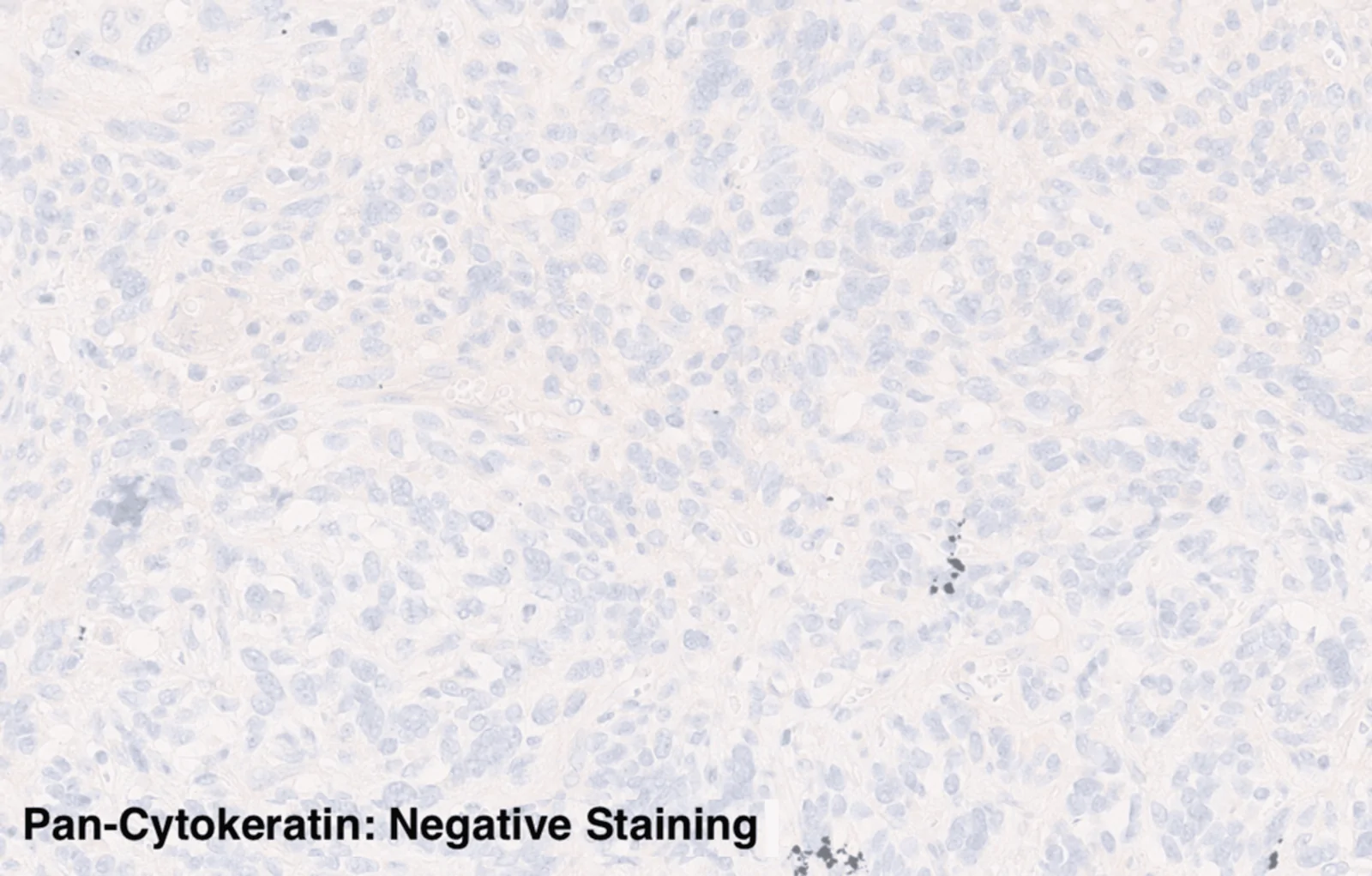
Tumor cells showing negative staining for pCK (IHC; 20×). pCK is considered the "Fingerprint” of Epithelial Tumors in Histopathology & Immunohistochemistry Staining. Therefore, "Negative Staining" of pCK rules out the possibility of the tumor being of epithelial origin. IHC: immunohistochemistry; pCK: pan-cytokeratin.

**Figure 6:**
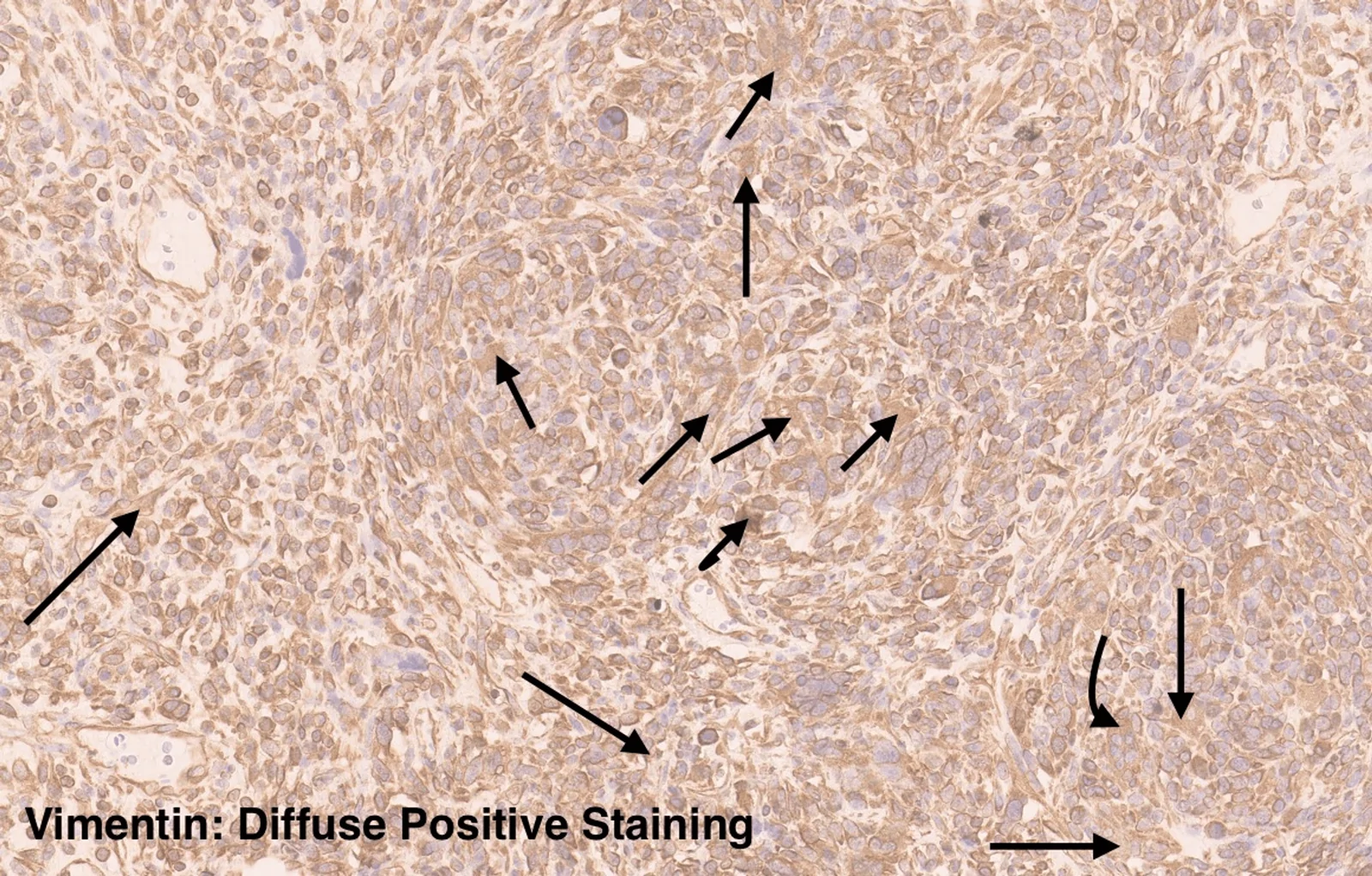
Tumor cells showing positive staining for vimentin (arrows) (IHC; 20×). Vimentin: A marker for "epithelial-mesenchymal transition (EMT)," which helps distinguishing sarcomas (which are vimentin-positive) from carcinomas. IHC: immunohistochemistry.

**Figure 7:**
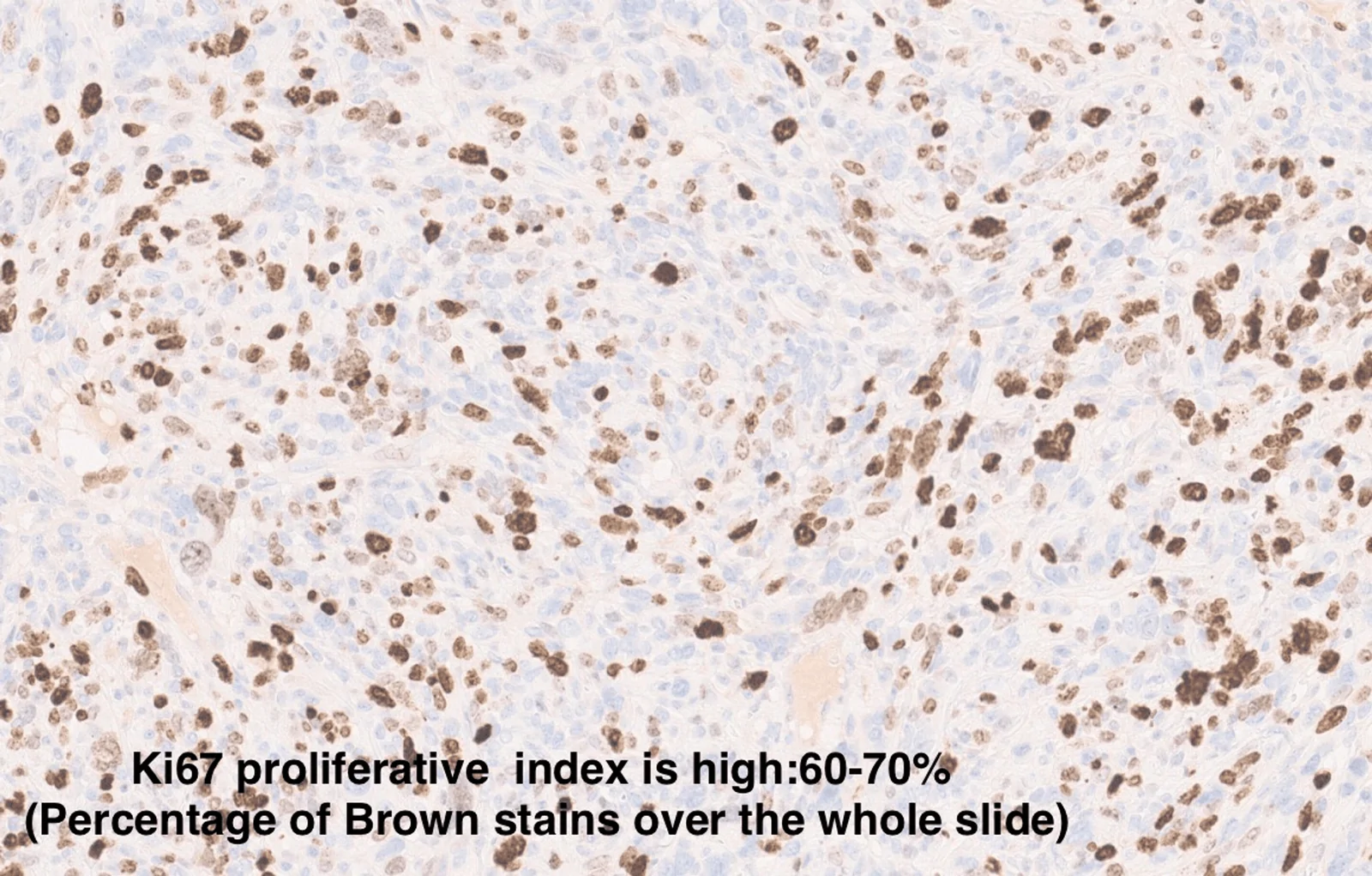
Ki-67 (a cellular marker for proliferation): tumor cells show a high proliferation index of approximately 60-70% (IHC; 20×).

The case was re-discussed in the MDT meeting for further adjuvant management; the consensus was "to discuss with the patient if she approves to receive a course of chemotherapy, as there is no solid evidence of benefit from adjuvant systemic therapy. Otherwise, the patient should start radiotherapy to chest wall operative bed due to the high risk of local recurrence." The patient signed for radiation but disagreed for chemotherapy. The patient has received the prescribed adjuvant radiation course (external beam radiotherapy to the chest wall), with a total dose of 60 Gy delivered over five weeks in 30 fractions of 200 cGy/fraction. Currently, she is under regular outpatient follow-up over the last two years, with no evidence of local recurrence or distant metastasis. She is planned for future autologous reconstruction using a latissimus dorsi myocutaneous flap. Figures 3-7 show the highlights of histopathological diagnosis and IHC.

## Discussion

Epidemiology and risk factors

Breast cancer is the most common cancer occurring in women worldwide. Most invasive breast tumors are epithelial in nature, whereas mesenchymal tumors are rare. PBS is extremely rare, with an estimated incidence of around 0.1% of all primary breast malignancies [3] and less than 5% of all soft tissue sarcomas. The estimated annual incidence is 4.6 per million women [4]. It is most commonly seen in women in their fifth or sixth decades of life [5]. The percentage of PBS in men is less than 5%.

To date, the exact etiology and pathogenesis of PBS remain undetermined. However, previous local radiation therapy and chronic lymphoedema may be associated with the development of secondary breast sarcoma. The latter typically occurs after external radiotherapy to either the breast or other intrathoracic cancers; the most common precedents are breast cancer and non-Hodgkin lymphoma [6]. In Li-Fraumeni syndrome, the development of breast sarcomas is strongly associated with TP53 mutation [7]. In addition, several environmental exposures were suggested as potential risk factors, including previous exposure to vinyl chloride, alkylating agents, and arsenic compounds [8].

Histopathology

There are different subtypes of PBS, and they are usually named after the type of their cell origin. The spectrum includes angiosarcoma, fibrosarcoma, osteosarcoma, chondrosarcoma, liposarcoma, leiomyosarcoma, rhabdomyosarcoma, malignant histiocytoma, and Kaposi's sarcoma. Angiosarcoma is the most common subtype [9].

Histopathological analysis is the cornerstone for the diagnosis of PBS along with an immunohistochemical panel, which is most valuable in ruling out malignant phyllodes tumors and metaplastic breast carcinoma of mesenchymal type, the two most important in a differential diagnosis [10]. Therefore, PBS is almost always diagnosed by excluding all mimickers, and distinguishing between them is of utmost importance for both treatment and prognosis. Phyllodes tumors have characteristic morphological features: a biphasic tumor (mixed fibroepithelial components), arranged in a leaf-like architecture, stromal overgrowth, and exaggerated intracanalicular pattern in the epithelial component. Metaplastic carcinoma is characterized on hematoxylin and eosin (H&E) sections by the presence of an epithelial carcinomatous component positive for cytokeratin or myoepithelial cells and transformation of epithelial component into non-glandular elements like spindle or squamous cells. The accurate diagnosis may be impossible to be confirmed with certainty by core biopsy, and in a significant portion of cases, a definitive diagnosis can only be achieved in the surgical resection specimen [11] as in our case.

IHC markers are not helpful in the subclassification of phyllodes tumors, but they are useful in differentiating other spindle cell lesions occurring in the breast. CD34 is expressed in the stroma of most phyllodes tumors [12]. Other markers include CD117 (patchy positivity), p53 (positive staining indicates high grade), CD10 (higher expression in malignant phyllodes), and low expression of ER/PR. For metaplastic carcinomas, usually triple-negative, the expression of cytokeratins (CKs) is variable and can be heterogenous within a tumor. Most cases are positive for CK5/6 and CK14, while CK7 and CK19 are less frequently positive. Of note, most metaplastic carcinomas are positive for myoepithelial antigen p63 [13].

Clinical presentation 

Breast sarcomas almost exclusively occur in women. However, very rare cases were reported in men: 2.4% of the cases investigated by Lim et al. and 1.5% in the data reported by Al-Benna et al. [14,15]. Breast sarcoma is typically diagnosed in the fifth or sixth decade of life, with a reported median age at diagnosis of 49.5 years. However, the age range is somehow wide (12-89 years). Patients diagnosed with secondary breast sarcomas are generally older than those with PBS probably because they tend to develop after treatment for breast cancer.

The usual presentation of breast sarcoma is a unilateral large-sized mass, well defined with a characteristic history of rapid increase in size, typically faster than a breast carcinoma. In two published studies, the median tumor size was 5.25 cm and 4.5 cm, with size ranges from 0.3 to 30 cm and from 0.1 to 48.4 cm, respectively [16]. Overlying skin discoloration may occur, but it is uncommon to find nipple changes, pain, or involved axillary lymph nodes.

Breast sarcomas have non-specific radiological findings. On mammography, the typical appearance is an opaque mass, but they rarely have spiculations or microcalcifications. On ultrasound, the mass is usually irregular with indistinct borders and no shadowing. MRI is frequently used to evaluate the extent of the disease and its relation to the overlying skin and underlying chest wall [17]. Again, the gold standard for the diagnosis of PBS is histopathological examination of a core biopsy, and in some cases, complete surgical excision may be needed to confirm the diagnosis, as was the case in this study.

Staging

The staging for PBS is different from that of breast carcinoma. It still follows the AJCC Cancer Staging Manual (revised 8th edition) soft tissue sarcoma of the trunk and extremities, which is based on tumor-node-metastasis (TNM) and tumor grade (G). The AJCC follows the grading system of the French Federation of Cancer Centers (FNCLCC) Sarcoma Group. Tumor grade is based on cellular differentiation, mitotic rate, and the extent of necrosis. Indeed, histology and grade are critical components of staging. Lymph node involvement is not characteristic of most sarcomas, except rhabdomyosarcoma and synovial, clear cell, vascular, and epithelioid sarcomas. All details are shown in Tables 1-4 [18,19]. After obtaining a tissue diagnosis of PBS, it is recommended to perform CT or MRI scan with contrast to evaluate the anatomical region of the tumor origin and obtain details about local extension. In addition, PET/CT scan is useful to detect distant metastasis.

**Table 1:** TNM classification for soft tissue sarcoma of the trunk and extremities. TNM: tumor-node-metastasis.

Primary tumor (T)	
TX	Primary tumor cannot be assessed
T0	No evidence for primary tumor
T1	Tumor ≤5 cm in greatest dimension
T2	Tumor >5 cm to ≤10 cm in greatest dimension
T3	Tumor >10 cm to ≤15 cm in greatest dimension
T4	Tumor >15 cm in greatest dimension
Regional lymph nodes (N)	
N0	No regional lymph node metastasis or nodes cannot be assessed
N1	Regional lymph node metastasis
Distant metastasis (M)	
M0	No distant metastasis
M1	Distant metastasis

**Table 2:** Basic classification for histologic grades.

Histologic grade (G)	
GX	Grade cannot be assessed
G1	Total differentiation, mitotic count and necrosis (score of 2 or 3)
G2	Total differentiation, mitotic count and necrosis (score of 4 or 5)
G3	Total differentiation, mitotic count and necrosis (score of 6, 7, or 8)

**Table 3:** Histologic grade (G) for soft tissue sarcoma. The scores for these variables are added to calculate the following G values: GX, grade cannot be assessed; G1, total score of 2 or 3; G2, total score of 4 or 5; G3, total score of 6 or higher. HPF: high-power fields; PNET: primative neuroectodermal tumor.

Tumor differentiation	Mitotic count	Tumor necrosis
Sarcoma closely resembling normal adult mesenchymal tissue (e.g., low-grade leiomyosarcoma) (1 point)	0-9 mitoses per 10 HPF (1 point)	No necrosis (0 points)
Sarcomas for which histologic typing is certain (e.g., myxoid/round cell liposarcoma) (2 points)	10-19 mitoses per 10 HPF (2 points)	<50% tumor necrosis (1 point)
Embryonal and undifferentiated sarcomas, sarcomas of doubtful type, synovial sarcomas, soft tissue osteosarcoma, Ewing sarcoma/PNET of soft tissue (3 points)	≥20 mitoses per 10 HPF (3 points)	≥50% tumor necrosis (2 points)

**Table 4:** Anatomic stage/prognostic groups. TNM: tumor-node-metastasis.

Stage	T	N	M	Histologic grade
Stage IA	T1	N0	M0	G1, GX
Stage IB	T2	N0	M0	G1, GX
	T3	N0	M0	G1, GX
	T4	N0	M0	G1, GX
Stage II	T1	N0	M0	G2, G3
Stage IIIA	T2	N0	M0	G2, G3
Stage IIIB	T3	N0	M0	G2, G3
	T4	N0	M0	G2, G3
Stage IV	Any T	N1	M0	Any G
	Any T	Any N	M1	Any G

Treatment

Surgery

The mainstay of treatment for PBS is surgical resection with adequately negative margins. For a long time, mastectomy without axillary lymph node dissection has been considered as the standard of care. However, the published survival analysis between mastectomy and breast-conserving surgery (BCS) did not establish a better prognosis with more radical treatment. Currently, the indications for BCS in PBS are somehow comparable to those in breast carcinoma. It is worth mentioning that the general recommendation for appropriate margin status in PBS is "more than 1 cm," which is different from that from breast carcinoma (no ink on tumor). Some studies recommended a margin of more than 3 cm for angiosarcoma and leiomyosarcoma [20,21]. In case of inability to achieve negative margins with satisfactory cosmetic results for large tumors, mastectomy should be the procedure of choice [22-24].

It is generally not recommended to perform axillary lymph node dissection or sentinel lymph node biopsy since PBS, as the case with any sarcoma, tends to spread by hematogenous route. The probability of lymphatic spread is below 5%, and a few unique cases were published [25,26]. In our case, due to uncertainty in preoperative tissue diagnosis and the discordance in axillary nodal status between clinical and radiological suspicious findings on one hand and the axillary fine needle biopsy on the other hand, we decided to perform sentinel lymph node biopsy (with the primary procedure), and it came negative by both frozen and paraffin sections.

*Adjuvant Therapy* 

The role of adjuvant therapy for PBS has been the subject of a heated debate. Some studies advocate the use of radiotherapy for large-sized tumors and high grades, at least 48 Gy to the tumor bed, arguing that it can improve disease-specific survival [27]. On the other hand, many others have stated that no benefits could be established [28,29]. The NCCN guidelines recommend considering adjuvant radiotherapy for cases with positive margins or higher stages. This can be as external beam radiation (50 Gy boost to tumor bed, 10-20 Gy in selected cases) or brachytherapy. In stage 2 and higher, preoperative radiotherapy could be considered yet still no published studies to confirm that.

Furthermore, there is no clear consensus about the role of adjuvant chemotherapy for PBS. Some articles recommended its use and linked both adjuvant systemic and radiation therapy to prolonged disease-free survival. However, some others failed to demonstrate a significant survival advantage from chemotherapy [30-32]. Also, there is some place for neoadjuvant chemotherapy (NAC) for PBS in published literature; one study reported its use in a patient with leiomyosarcoma fixed to the chest wall. After NAC, the tumor became mobile, and surgery was performed [33,34].

Prognosis

PBS is a rare and aggressive tumor. The average five-year overall survival is approximately 50-66%, with a disease-free survival of 33-52%. Notably enough, PBS has a poor prognosis compared to malignant phyllodes. Based on the available literature, the most significant prognostic factor is obtaining satisfactory negative margins during surgery as this is linked to the risk of local recurrence and survival [35]. Other recognized factors include age, tumor size (poor prognosis if larger than 5 cm), tumor grade, distant metastasis, nodal disease, radiation history, and histological subtype. Angiosarcoma has a poor prognosis compared to other sarcoma subtypes [36].

## Conclusions

Breast sarcomas are rare mesenchymal tumors arising from the connective tissue in breast parenchyma. It should be noted that breast sarcomas (non-epithelial) are different from malignant phyllodes tumors (fibroepithelial) and have poorer prognosis. They can be primary (de novo) or secondary to previous local radiotherapy. PBS is a particularly rare and aggressive entity with a poor prognosis and no specific guidelines designed for. Diagnosis is typically by triple assessment, and some cases may need to proceed with complete surgical excision to confirm the diagnosis. The management in general follows the guidelines for soft tissue sarcoma of the trunk and extremities, but it is necessary to be tailored on a case-by-case basis and supported by MDT consensus. The mainstay of treatment is surgery with satisfactory negative margins (>1 cm for all sarcomas and >3 cm in angiosarcoma and leiomyosarcoma). To date, there is no solid consensus about adjuvant or neoadjuvant therapy in general but can be of use in stage 2 and higher. More research is needed to obtain a better understanding and achieve an optimum level in the management of PBS.
